# Biological aging of different blood cell types

**DOI:** 10.1007/s11357-024-01287-w

**Published:** 2024-07-26

**Authors:** Saara Marttila, Sonja Rajić, Joanna Ciantar, Jonathan K. L. Mak, Ilkka S. Junttila, Laura Kummola, Sara Hägg, Emma Raitoharju, Laura Kananen

**Affiliations:** 1 Molecular Epidemiology (MOLE), Faculty of Medicine and Health Technology, Tampere University, Tampere, Finland; 2 Gerontology Research Center, Tampere University, Tampere, Finland; 3 Tays Research Services, Wellbeing Services County of Pirkanmaa, Tampere University Hospital, Tampere, Finland; 4 Department of Medical Epidemiology and Biostatistics, Karolinska Institute, Stockholm, Sweden; 5 Department of Pharmacology and Pharmacy, Li Ka Shing Faculty of Medicine, The University of Hong Kong, Hong Kong, China; 6 Faculty of Medicine and Health Technology, Tampere University, Tampere, Finland; 7 Fimlab Laboratories, Tampere, Finland; 8 Northern Finland Laboratory Centre (NordLab), Oulu, Finland; 9 Research Unit of Biomedicine, University of Oulu, Oulu, Finland; 10 Faculty of Social Sciences (Health Sciences), Tampere University, Tampere, Finland; 11 Department of Neurobiology, Care Sciences and Society (NVS), Karolinska Institute, Stockholm, Sweden

**Keywords:** Biological age, Epigenetic clock, Telomere length, DNA methylation, Biological aging, Blood cell subtypes

## Abstract

Biological age (BA) captures detrimental age-related changes. The best-known and most-used BA indicators include DNA methylation–based epigenetic clocks and telomere length (TL). The most common biological sample material for epidemiological aging studies, whole blood, is composed of different cell types. We aimed to compare differences in BAs between blood cell types and assessed the BA indicators’ cell type-specific associations with chronological age (CA). An analysis of DNA methylation–based BA indicators, including TL, methylation level at cg16867657 in *ELOVL2*, as well as the Hannum, Horvath, DNAmPhenoAge, and DunedinPACE epigenetic clocks, was performed on 428 biological samples of 12 blood cell types. BA values were different in the majority of the pairwise comparisons between cell types, as well as in comparison to whole blood (*p* < 0.05). DNAmPhenoAge showed the largest cell type differences, up to 44.5 years and DNA methylation-based TL showed the lowest differences. T cells generally had the "youngest" BA values, with differences across subsets, whereas monocytes had the "oldest" values. All BA indicators, except DunedinPACE, strongly correlated with CA within a cell type. Some differences such as DNAmPhenoAge-difference between naïve CD4 + T cells and monocytes were constant regardless of the blood donor's CA (range 20–80 years), while for DunedinPACE they were not. In conclusion, DNA methylation–based indicators of BA exhibit cell type–specific characteristics. Our results have implications for understanding the molecular mechanisms underlying epigenetic clocks and underscore the importance of considering cell composition when utilizing them as indicators for the success of aging interventions.

**Supplementary Information:**

The online version contains supplementary material available at 10.1007/s11357-024-01287-w.

## Introduction

By definition, biological age (BA), or an aging biomarker, should predict future health status better than chronological age. According to AFAR (American Federation for Aging Research) criteria, “It must monitor a basic process that underlies the aging process, not the effects of disease.” [1–4] Of the many established BA indicators [4, 5], the best-known and most-used are DNA-methylation-based epigenetic ages (epigenetic clocks) and telomere length, a hallmark of aging [6]. Ideally, these indicators should reflect the influence that health interventions have on biological aging. However, the underlying molecular mechanisms of the epigenetic clocks remain unknown.

Accelerated biological aging (or aging rate), indicated by telomere length [7] and epigenetic clocks [6], predicts the health span, lifespan, or both in large-scale cohort studies, with epigenetic clocks outperforming telomere length [4, 8]. Typically, these analyses are performed with whole blood samples that are mixtures of various blood cell subtypes. As such, the blood cell composition is a potential confounder in the analyses because it changes with advancing age [9], starting from before middle age [10]. Typical characteristics of the age-related remodeling of the immune system include decreasing naïve CD8 and CD4 T cell counts and increasing exhausted CD28- T cell counts, a declining CD4 to CD8 T cell ratio, and potentially also an altered NK cell count and functionality [11, 12]. This remodeling is highlighted by the existence of the IMM-AGE [13], a blood cell composition–based, potential BA indicator. Furthermore, changes in blood cell composition are seen in many age-related conditions (e.g., frailty [14]) and diseases, such as cancer [15], Alzheimer’s disease [16], and cardiovascular diseases [17, 18]. The age-related remodeling of the blood cell composition is not limited to these changes, but these are the well-known examples for which there is epidemiological evidence regarding their relationship to aging and aging phenotypes.

A better understanding of biological aging at the cell subtype–level within tissues is needed. Previous studies have shown that telomere length [19–22] and DNA methylation level at cg16867657, a CpG site in *ELOVL2* [23], are tissue and cell type–specific in their absolute values and age-related changes. A few previous studies have shown that epigenetic ages determined by DNAmPhenoAge and Horvath differ between blood cell types [24], or that cell proportions correlate with these [25, 26], but the BA or biological aging rate indicated by more recently developed epigenetic clocks have been studied less in separated cell subtypes. Importantly, such previous analyses have typically been made using separated cells originating from different individuals and datasets with less than 10 individuals each [27]. Furthermore, the way in which cell subtype–specific epigenetic age values indicated by the 2nd and 3rd generation epigenetic clocks change with advancing chronological age across adulthood remains unknown. Thus, in this study, we aimed to **1)** assess differences in values of DNA methylation–based BA indicators between blood cell types originating from the same blood donors and with a more adequate sample size. We also aimed to **2)** assess the BA indicators’ cell type–specific associations with the donor’s chronological age. The BA indicators included the “1st generation clocks” (ELOVL2-CpG-site, cg16867657 [23], Horvath [28], and Hannum [29]), the “2nd generation clock” (DNAmPhenoAge [30]), the “3rd generation clock” (DunedinPACE [31]), as well as telomere length (DNAmTL, estimated based on DNA methylation data [32]). In our main analyses, we performed pairwise comparisons of BA indicator values between whole blood, peripheral blood mononuclear cells (PBMCs), and up to ten separated blood cell subtypes in four separate datasets with 428 biological samples, originating from the same blood donors. Then, we assessed the cell subtype–specific associations of the different BA indicators with chronological age. In our additional analyses, we repeated pairwise comparison analyses with the principal component derivates of the clocks [33], assessed cell subtype–specific correlations between the different BA indicators, and, lastly, illustrated examples of blood cell subtype count trajectories over decades in a longitudinal cohort sample (The Swedish Adoption/Twin Study of Aging [SATSA], n = 328).

## Methods

### Datasets

In the present study, we included four datasets available in the NCBI GEO [34, 35] (GSE35069 [36], GSE131989 [37], GSE166844 [24], and GSE78942 [38]), in which DNA methylation data were available from separated immune cell subtypes (Table 1). These subtypes were separated using fluorescence-activated cell sorting (FACS), as described in detail in the original publications [24, 36–38]. The surface markers used for the FACS analyses are summarized in Supplementary Table S1. We included only datasets in which the different immune cell populations were available from the same individuals as complete cases. For the cell count trajectory analysis, DNA methylation–based cell count estimates of whole blood samples included in the Swedish Adoption/Twin Study of Aging (SATSA, n = 328, with 657 observations, baseline ages 48–98, mean age 68.5) were used [39].

**Table 1:** Datasets in pairwise comparisons

Dataset	Individuals, *n*	Chronological age (years)	Chronological age available for each individual	Female, %	Cell sample types, *n*	Available cell sample types	Pairwise cell type comparisons, *n*
GSE131989	49	54 ± 17.5	Yes	100	4	CD14 + , CD19 + , CD4 memory T cells, CD4 naïve T cells	6
GSE166844	28	19 ± 0	Yes	40	6	Whole blood, Granulocytes, CD14 + monocytes, CD19 + B cells, CD8 + T cells, CD4 + T cells	15
GSE35069	6	38 ± 13.6	No	0	10	Whole blood, PBMC, Granulocytes, Neutrophils, Eosinophils, CD14 + monocytes, CD19 + B cells, CD56 + NK cells, CD8 + T cells, CD4 + T cells	45
GSE78942	24*	62.1 ± 9.9	No	NA	2	CD4 + CD28 + T cells, CD4 + CD28- T cells	1

*DNA methylation measured from two pooled samples of separated cells; 12 individuals per sample

### BA indicators

We assessed different BA indicators using DNA methylation data from the aforementioned datasets (Table 1). The investigated indicators of BA (or biological aging rate) were telomere length estimated based on DNA methylation (DNAmTL) [32], the methylation level of *ELOVL2* at one CpG (cg16867657) [40], as well as Hannum [29], Horvath [28], DNAmPhenoAge [30], and DunedinPACE [31], in addition to the principal component derivates of DNAmTL, Hannum, Horvath, and DNAmPhenoAge [33]. In three of the included datasets, DNA methylation was measured using an Illumina 450 K (GSE35069, GSE13198) or Illumina EPIC (GSE166844) array, allowing us to calculate all ten indicators of BA. In dataset GSE78942, methylation data were measured using an Illumina 27 K array, allowing us to calculate only Horvath and DNAmPhenoAge values. All BA indicators were calculated from the normalized and preprocessed data available in the GEO.

The DNAmTL, Hannum, Horvath (for datasets GSE35069, GSE13198, and GSE166844) and DNAmPhenoAge (for datasets GSE35069, GSE13198, GSE166844 and GSE78942) were calculated using R software and the DNAmAge function of the methylclock R package version 0.8.2 [41]. For GSE78942, the Horvath was calculated using the online tool available at https://dnamage.clockfoundation.org/. DunedinPACE was calculated as described in the original publication [31] with the R package DunedinPACE. The principal component derivates of the clocks were calculated as described previously [33]. The methylation value of the probe cg16867657 in *ELOVL2* was extracted directly from methylation data available in the GEO for each dataset.

### Statistical analysis

Statistical significance for the pairwise comparisons was assessed using the Mann–Whitney U test. BA values were compared between the cell subtypes at group level within a dataset. Cell subtype–specific BA values were visualized as boxplots with dots and line plots, and pairwise differences as boxplots. Cell subtype–specific relationships between the values of different BA indicators and chronological age were assessed using correlation statistics (Spearman), and the relationships were visualized as scatterplots.

In our additional analyses, we also applied correlation statistics (Spearman) to assess the cell subtype–specific relationships between the values of different BA indicators and visualized the relationships with scatterplots. In the longitudinal cohort data, cell subtype count trajectories were visualized as line plots, and the significance of the cell count change with chronological age was obtained using a mixed linear model. In GSE131989 and SATSA, chronological age was used as individual-level phenotypic data in our statistical analyses. Data were analyzed and visualized using R statistical software and the R package ggplot2. The *p*-value threshold for statistical significance was set to 0.05.

Details on how different cell types were separated are provided in Supplementary Table 1.

## Results

### Pairwise comparisons

**Fig. 1:**
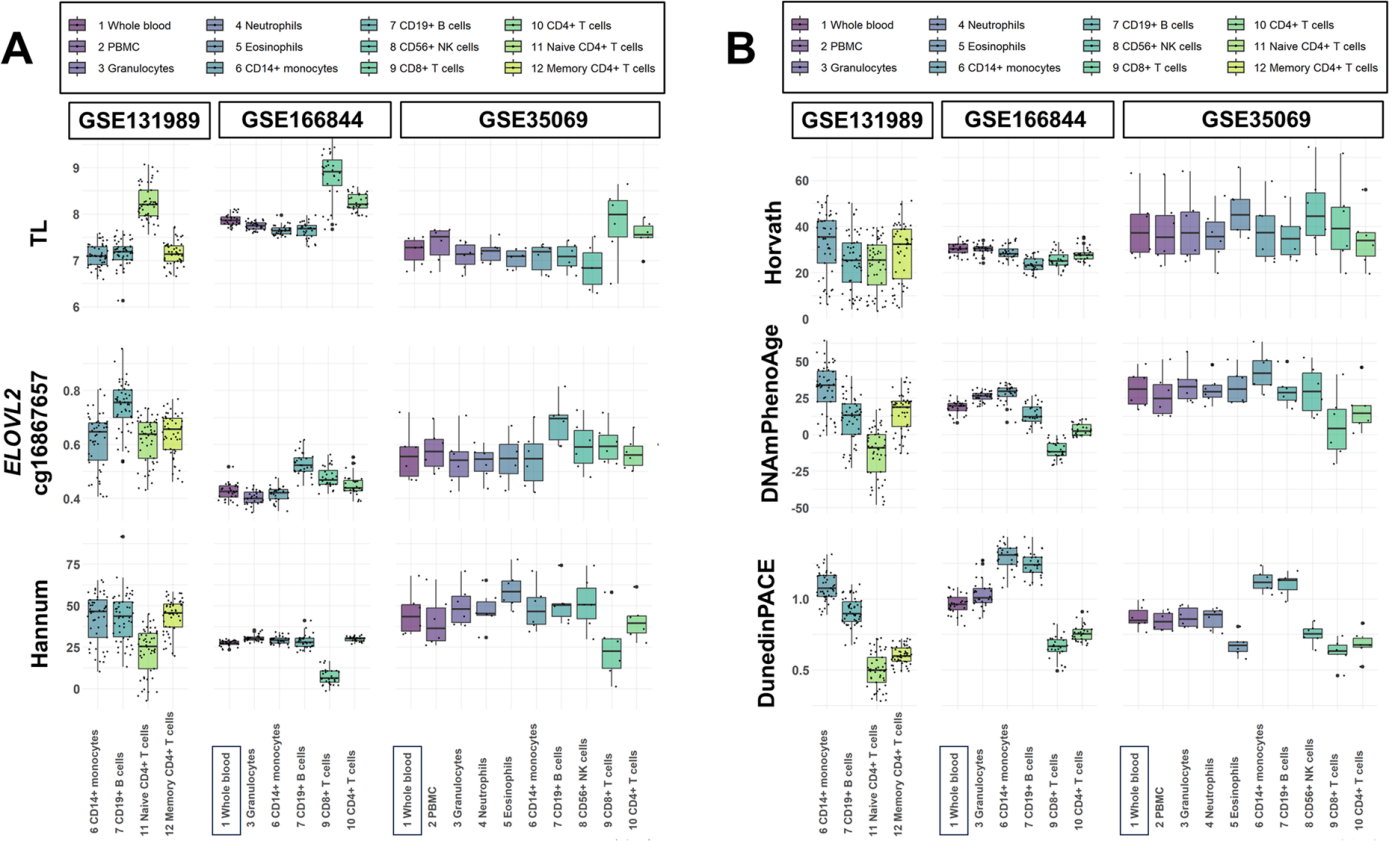
Blood cell type–specific biological ages (BAs) and a BA rate

**Fig. 2:**
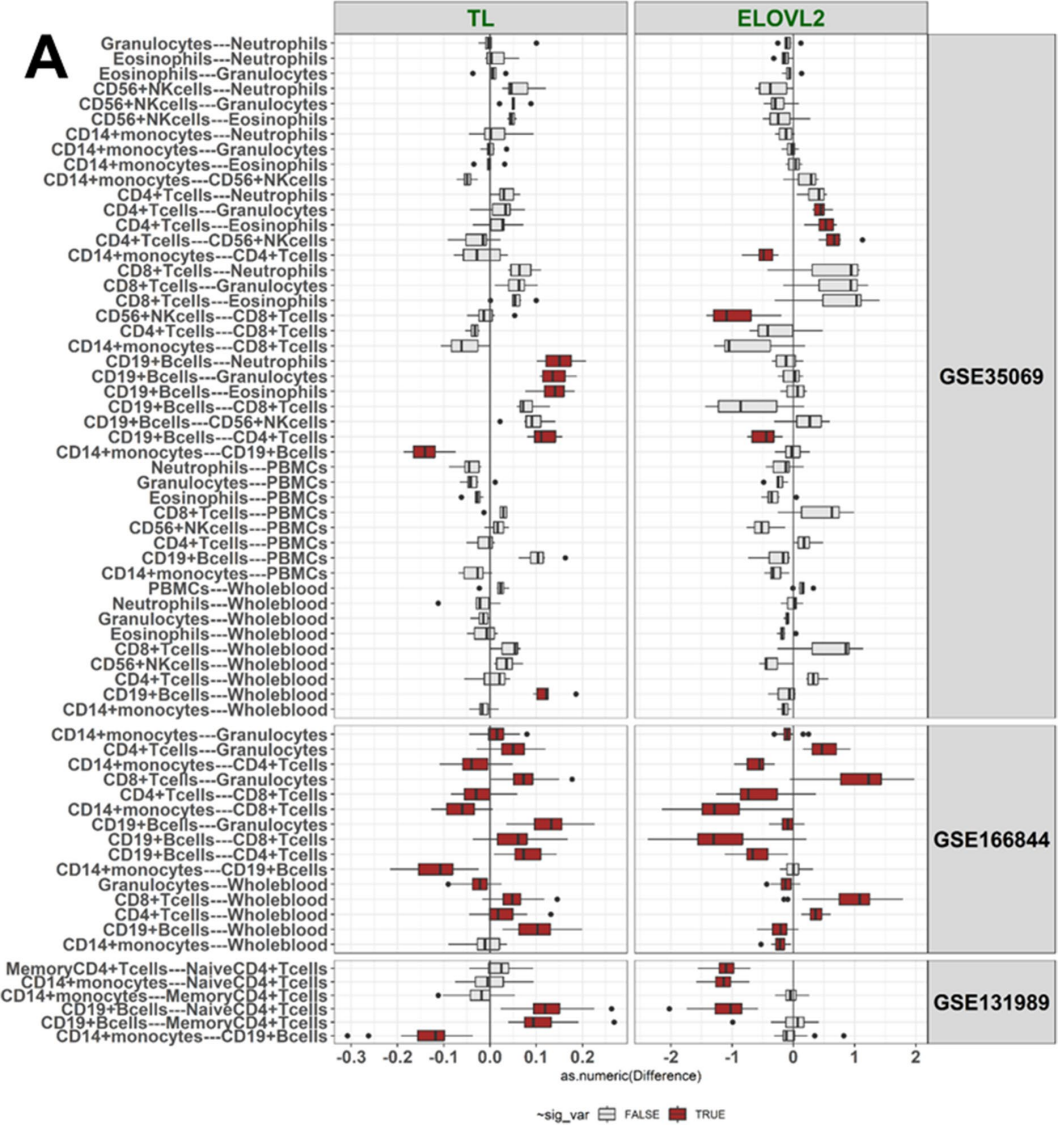

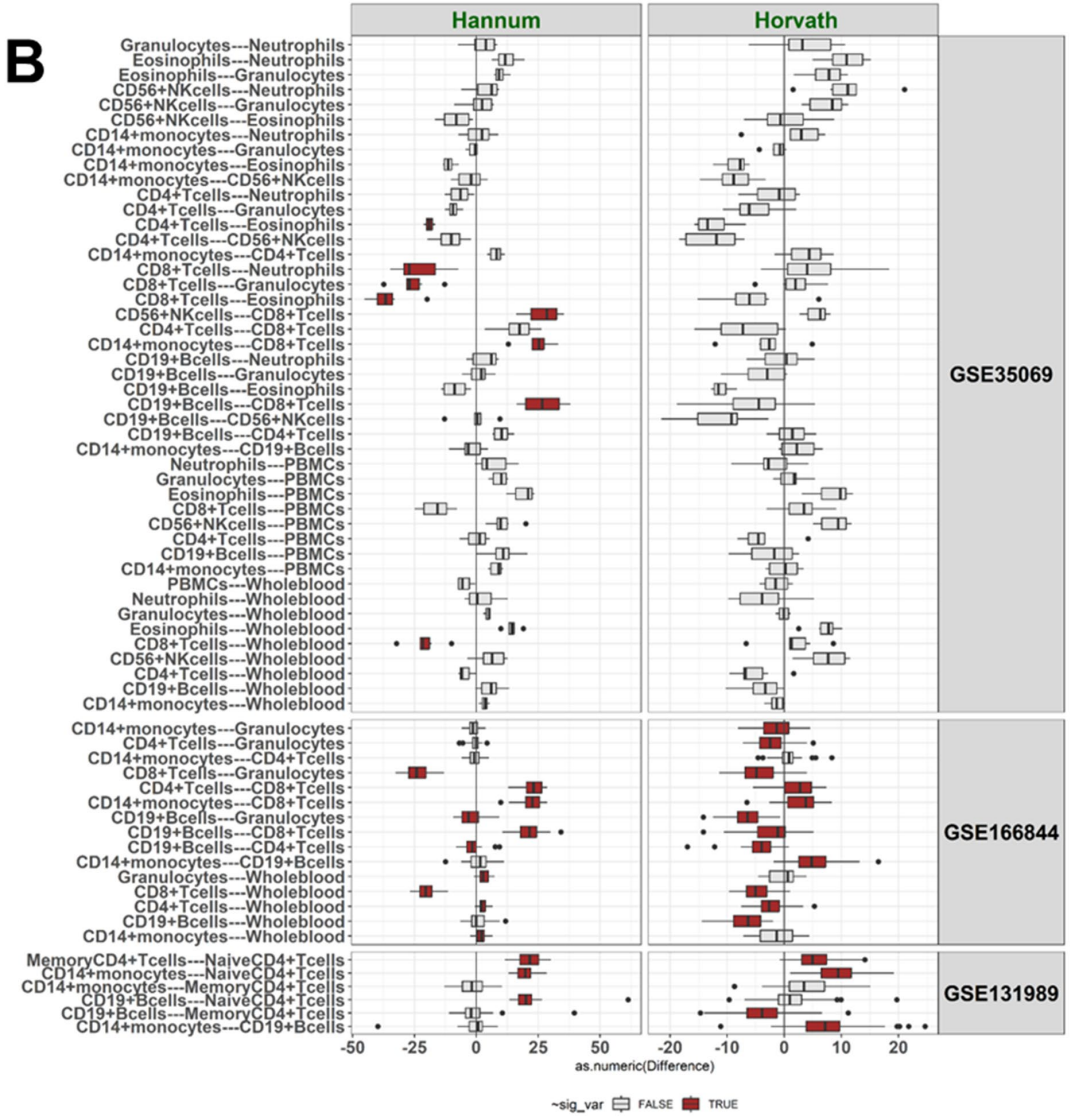

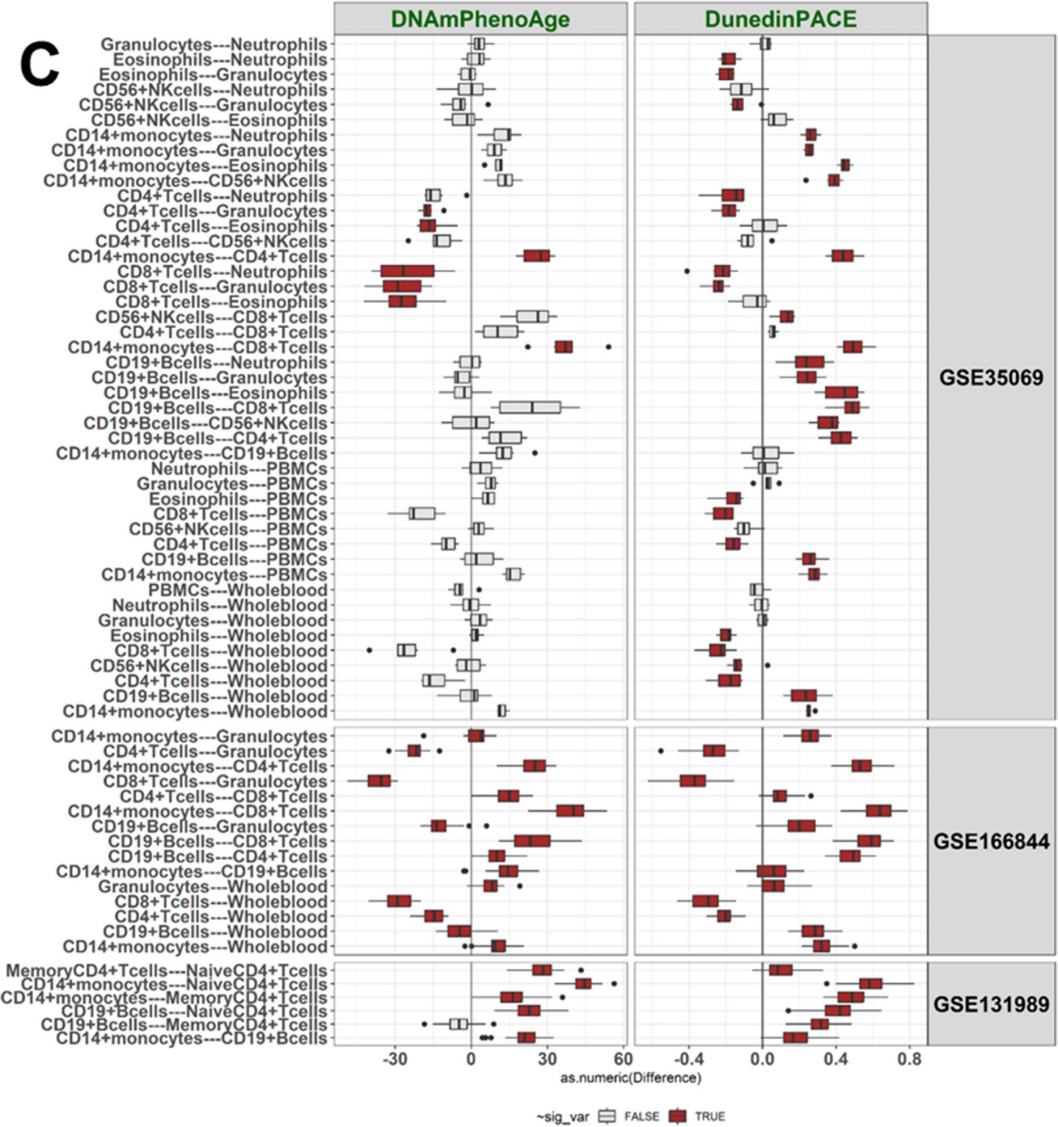
Pairwise differences in values of cg16867657 at *ELOVL2* (ELOVL2) and DNAmTL (TL) (**A**), in Hannum and Horvath values (**B**), and in DNAmPhenoAge and DunedinPACE values (**C**) between the cell types

**Fig. 3:**
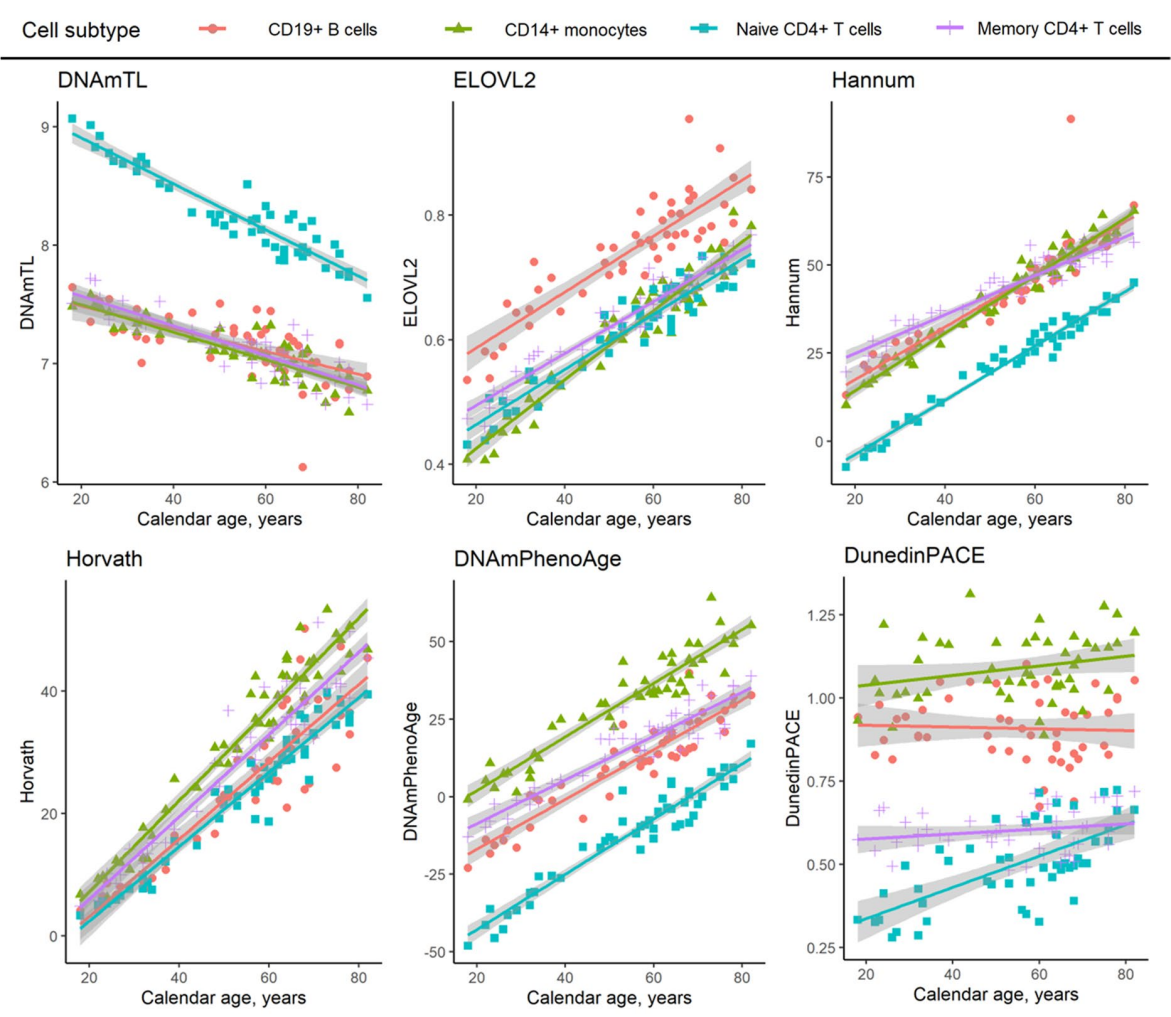
Cell type–specific associations of DNA methylation–based BA indicators (and biological aging rate) with blood donors’ chronological age in GSE131989

The BA values for each cell type in the different datasets (Table 1) included in our analysis are shown in Fig. 1, Table 2, Supplementary Figure S1, and Supplementary Table S2. As our main analysis, we performed pairwise comparisons of the BA indicator values between the blood cell subtypes. In summary, BA values, including the principal component derivates of the epigenetic clocks, were different (Mann–Whitney U test p < 0.05) in the majority of the pairwise comparisons between the cell types (Table 2, Fig. 2, Supplementary Table S3–S7, Supplementary Results). Most cell types also displayed differences when compared to whole blood (Mann–Whitney U test p < 0.05, Fig. 2, Supplementary Tables S3–S7). Some of the observed differences persisted across the blood donors’ chronological ages of 20–80 years; for example, the 50-year difference in DNAmPhenoAge values between naïve CD4 + T cells and monocytes (Fig. 3). However, the up to four-fold difference in DunedinPACE values between monocytes and B cells, for instance, did not persist over time (Fig. 3).

**Table 2:** BA values according to Horvath and DNAmPhenoAge displayed “younger” values in CD4 + CD28 + T cells compared to CD4 + CD28- T cells in GSE78942

Dataset	Sample type, sample ID	Horvath	DNAmPhenoAge
**GSE78942**	**CD4 + CD28- T cells, pool1**	65.9	50.8
	**CD4 + CD28- T cells, pool2**	72.9	57.0
	**CD4 + CD28 + T cells, pool1**	39.0	8.5
	**CD4 + CD28 + T cells, pool2**	35.1	10.7

As expected, the pairwise comparisons were more often statistically significant (Mann–Whitney U test p < 0.05) in the GSE166844 and GSE131989 datasets with larger numbers of individuals than in GSE35069, which included six individuals (Tables 1 and 3). Further important details as regards the interpretation of the results are that GSE131989 and GSE35069 included individuals with a wide chronological age range, while the individuals in GSE166844 were all 19 years old. Most cell types were available in at least two datasets, but neutrophils, eosinophils, and CD56 + NK cells were only available for analysis in GSE35069. In GSE78942, the difference in BA values was apparent, but statistical analysis was not possible, as it comprised only four biological samples.

**Table 3:** Number of cell pairs showing differences for each indicator of BA in three datasets

	Dataset		
	**GSE131989**	**GSE166844**	**GSE35069**
**Number of pairwise comparisons**			
	**6**	**15**	**45**
**Number of cell pairs showing pairwise differences (%)**			
**DNAmTL**	3 (50)	14 (93)	6 (13)
**ELOVL2**	3 (50)	14 (93)	6 (13)
**Hannum**	3 (50)	10 (67)	8 (18)
**Horvath**	4 (67)	12 (80)	0 (0)
**DNAmPhenoAge**	5 (83)	15 (100)	7 (16)
**DunedinPACE**	6 (100)	15 (100)	31 (69)

DNAmTL (TL), cg16867657 in *ELOVL2*, and Hannum values are summarized as boxplots with dots in panel A, and Horvath, DNAmPhenoAge, and DunedinPACE values in panel B. These DNA methylation–based BA indicators were assessed in three DNA methylation datasets (GSE131989, GSE166844, and GSE35069) with 424 biological samples from 83 individuals and including 12 cell sample types. Boxes are colored according to cell type (1–12). Each smaller dot represents one individual.

DNA methylation was measured from four pooled biological samples of purified cells. In pool1, cells were separated from 12 individuals and pooled as two biological samples (CD28 + and CD28- cells). In pool2, cells originated from another set of 12 individuals and the separated cells were pooled in a manner similar to pool1. The chronological age of these healthy blood donors was 45–75 years (mean[SD] = 62.1[9.9]).

Cell pairs with a statistically significant difference in BA values (Mann–Whitney U, p < 0.05) are colored in red, others in grey. The difference in BA indicator values between a cell pair (Δ) was calculated for each individual, and these differences are shown as boxplots for the three datasets. The Δ-value for a BA indicator is shown on the x axis. The GSE131989 dataset with 49 blood donors had 6 cell type pairs, the GSE166844 with 28 blood donors had 15 pairs, and the GSE35069 with six blood donors had 45 cell type pairs to be compared. Cell type–specific BA values within a dataset are summarized in Fig. 1, Supplementary Fig. 1, and Supplementary Table S2, and *p*-values for the comparisons are presented in Supplementary Tables S3–S5.

Mann–Whitney U test *p*-values are shown in Supplementary Tables S3–S5.

DNA methylation data within four separated cell subtypes (CD19 + B cells, CD14 + monocytes, naïve CD4 + T cells, and memory CD4 + T cells) originated from 49 individuals aged 18–82 years (all females). Correlation statistics showing the significance for the associations with chronological age are shown in Supplementary Table S8. Grey areas indicate 95% Confidence Intervals for the linear fit lines.

### CD19 + B cells

Methylation data for CD19 + B cells were available in three datasets. In comparison to other cell types, CD19 + B cells displayed a statistically significant difference (Mann–Whitney U test *p*-value < 0.05) in the majority of the pairwise comparisons in GSE131989 and GSE166844 (Fig. 2, Supplementary Tables S3–S4). In the smallest dataset, GSE35069, statistically significant differences were mainly observed for DunedinPACE (Fig. 2, Supplementary Figure S1, Supplementary Table S5). In summary, our results suggest that CD19 + B cells are, according to the studied BA indicators, “younger” than CD14 + cells, but “older” than naïve CD4 + cells and total CD8 + T cells, although there are some discrepancies between the different BAs (Fig. 2, Supplementary Tables S3–S5). In comparison to whole blood, no clear pattern was observed for CD19 + B cells.

### T cell subsets

Data on various subsets of T cells were available in four datasets, including total CD4 + and CD8 + T cells (GSE166844 and GSE35069), CD4 + naïve and memory T cells (GSE131989), as well as CD4 + CD28- and CD4 + CD28 + T cells (GSE78942). The majority of the pairwise comparisons across these cell types were statistically significant (Fig. 2, Table 2, Supplementary Tables S3–S5). Our results suggest that CD8 + T cells are “younger” than CD4 + T cells and that naïve CD4 + T cells are “younger” than memory CD4 + T cells (Fig. 2, Supplementary Tables S3–S5). In addition, CD4 + CD28 + cells were identified to be “younger” than CD4 + CD28- cells according to both BA indicators available for this dataset, Horvath and DNAmPhenoAge (Table 2), although no statistical tests could be performed on these data, as there were only four biological samples. In comparison to whole blood, both CD4 + and CD8 + T cells are “younger”, although there are discrepancies between the different BA indicators (Fig. 2, Supplementary Tables S3–S5). The magnitude of the difference was larger between CD8 + T cells and whole blood than between CD4 + T cells and whole blood (Fig. 1, Fig. 2, Supplementary Table S7).

### CD14 + monocytes

Data on CD14 + monocytes were available in three datasets. In comparison to other cell types, the majority of the pairwise comparisons between CD14 + monocytes were statistically significant in GSE166844 and GSE131989 (Fig. 2, Supplementary Figure S1, Supplementary Tables S3 and S4). Our results suggest that CD14 + monocytes are “older” than various T cell subsets, in addition to being “older” than CD19 + B cells as well as whole blood samples (Fig. 2, Supplementary Tables S3–S5).

### Magnitude of differences

We identified differences across cell types for all six BA indicators (Table 3), but the magnitude of the differences varied (Fig. 2). For example, of the epigenetic clocks measured in years (*i.e.* Hannum, Horvath and DNAmPhenoAge), the largest differences were observed for DNAmPhenoAge (on average 11–23 years across all cell comparisons in GSE131989, GSE166844 and GSE35069, Supplementary Table S6), whereas the differences were the smallest for Horvath (from three to five years across datasets, Supplementary Table S6). The maximum difference among the three clocks was observed for DNAmPhenoAge values, between CD14 + monocytes and naïve CD4 + T cells (44.5 years, across all cell comparisons and data sets, Supplementary Table S6). The largest difference in Horvath values (nine years) was observed between CD14 + monocytes and naïve CD4 + T cells whereas for Hannum, the maximum difference (37 years) was between CD8 + T cells and eosinophiles (Fig. 2, Supplementary Table S6).

Then, to compare all six BA indicators with each other, not just the ones measured in years, we calculated the difference in percentages for each cell type as compared to the whole blood sample (Supplementary Table S7). Whole blood data was available in GSE35069 and GSE166844, and, on average, the largest differences in percentages were observed for DNAmPhenoAge (24% and 75%) and the smallest for DNAmTL (4% and 5%) in the two data sets. The differences for *ELOVL2* methylation (6% and 9%) were the second smallest across the BA indicators in the two datasets (Supplemetary Table S7). The percentage differences of Hannum (18% and 21%) and DunedinPACE (17% and 24%) were smaller than those of DNAmPhenoAge (24% and 75%) but larger than of Horvath (8% and 12%, in GSE35069 and GSE166844, respectively, Supplementary Table S7).

### Cell subtype–specific BA values across adult chronological ages

All BA indicators, except for DunedinPACE, correlated strongly with chronological age within a cell type in dataset GSE131988 (> 0.8 or < -0.7, Fig. 3, Supplementary Table S8). DunedinPACE values increased most consistently with increasing chronological age for naïve CD4 + T cells (Spearman’s ρ = 0.636), but for the other cell types tested, the correlations were more modest or non-existent (Fig. 3, Supplementary Table S8). The analysis was performed in this one dataset, as either the chronological age was not available, or all individuals were of the same age in the other datasets.

### Additional analyses

#### Pairwise comparisons for principal component clocks

The epigenetic clocks have been reported to suffer from technical noise [33]. The proposed solution is to utilize principal components instead of the individual-level CpG data to calculate the clocks, i.e. PC clocks. As an additional analysis to verify that the observed differences in BA indicators across cell types are not due to technical noise of the Illumina array, we repeated the pairwise comparison analysis with the principal component derivates for DNAmTL, Hannum, Horvath, and DNAmPhenoAge (Supplementary Table S2). Our results show that the observed differences between the cell types in the main analysis remained significant for the studied PC clocks (Supplementary Tables S3- S5).

#### Relationships between different BAs

We then explored the relationships between the values of different BA indicators (Supplementary Figure S2) and focused on the relationships within each cell subtype population (Supplementary Table S8–S10). The majority of the BA indicators showed strong or very strong correlations (> 0.7 or < -0.7) with each other within the different cell subtype populations in GSE131989 and GSE35069 (Supplementary Table S8 and S10), which have wide age ranges. However, very few moderate or stronger correlations (> 0.5 or < -0.5) were observed in GSE166844 (Supplementary Table S9), which includes only individuals of the same chronological age. An exception in the cell type–specific correlations was seen for DunedinPACE where the correlations were, overall, lower or non-existent (Fig. 3A, Supplementary Tables S6–S8).

#### Blood cell composition trajectories

In the last additional analysis, we visualized estimated blood cell composition trajectories in a longitudinal cohort (SATSA) with decades of follow-up (Supplementary Figure S3) and observed changes in cell counts with advancing chronological age for all blood cell subtypes that were included in our pairwise comparisons and also available in SATSA (*p* < 0.005). The counts of B cells, CD4 + and CD8 + T cells, as well as naïve CD4 + and CD8 + T cells decrease, while the counts of CD8 + CD28-CD45RA- T and NK cells, plasmablasts, monocytes, and granulocytes increase from midlife into old age (Supplementary Figure S3).

## Discussion

We assessed ten DNA methylation–based BA indicators— DNAmTL [32], Hannum [29], Horvath [28], DNAmPhenoAge [30], and their principal component derivates [33], as well as DunedinPACE [31] and the methylation level of *ELOVL2* at cg16867657 [40]—in 428 biological samples, in up to 12 blood cell types, collected and separated from the same set of individuals. Our results show a significant difference (p < 0.05) in BA values, including the principal component derivates of the epigenetic clocks, in the majority of the pairwise comparisons between the cell types and in comparison to whole blood. These pairwise differences were most prevalent for DunedinPACE. The largest differences were observed for DNAmPhenoAge, up to 44.5 years, whereas DNAmTL was most consistent across the cell types. The difference between whole blood cell sample and other cell subtypes can be substantial, up to 160% for DNAmPhenoAge, and on average the differences were 20 percent or more for Hannum, DNAmPhenoAge and DunedinPACE, 10 percent for Horvath and less than 10 percent for DNAmTL and *ELOVL2* methylation.

**Fig. 4:**
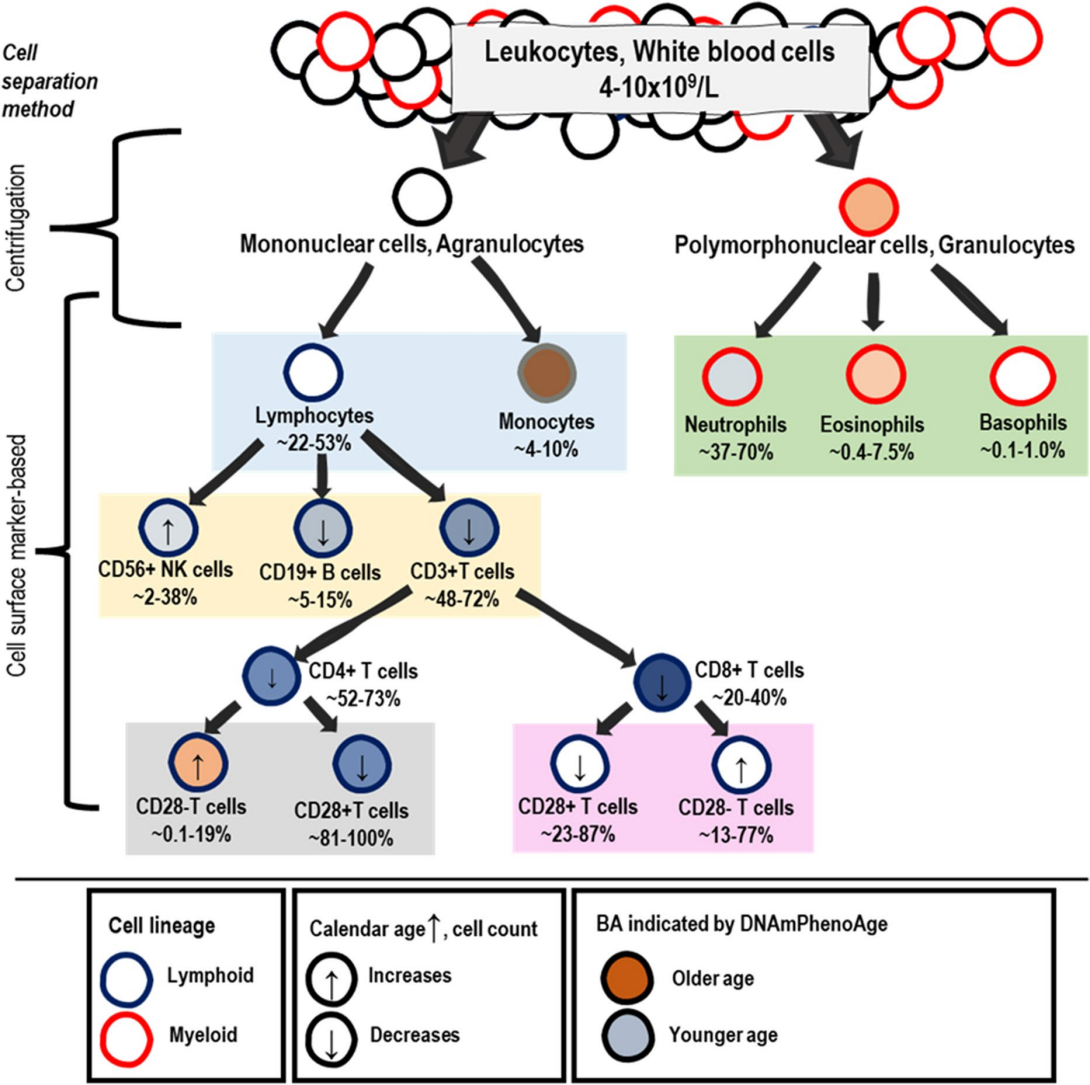
Graphical summary of typical blood cell subtype separation with cell type–specific biological ages (BAs), as well as cell proportion ranges and age-related changes at the human population level

As a new finding, we show that the cell type–specific BA values of the blood cells appear to persist across human adulthood, with the exception of DunedinPACE. For example, the 50-year difference in DNAmPhenoAge values between naïve CD4 + T cells and CD14 + monocytes persists across chronological ages from 20 to 80 years. To put the 50-year difference into perspective, the BA value difference is approximately 60 years between a 20-year-old and an 80-year-old person, but the cell type–specific difference is a few years between two persons of the same chronological age. Thus, in line with Zhang et al. (2023) [27], we conclude that chronological age and blood cell composition together explain the great majority of variation in BA values. As an exception among the BA indicators, the DunedinPACE values can show up to four-fold differences between the cell types, but the differences do not appear to persist throughout the human lifespan across all of the cell types studied herein. Furthermore, by using longitudinal cohort data, we highlight how thoroughly the blood cell composition changes with age during adulthood, which is in line with previous reports [13, 26, 42–48]. The synthesis of this evidence implies that the proportion of many of the cell types with “younger” BA values in blood circulation, such as naïve CD4 + and naïve CD8 + T cells, declines with advancing chronological age, while cells with mostly “older” BA values, such as monocytes, become more prevalent (Fig. 4).

Cell count prevalence ranges and changes with advancing chronological age at population level are presented according to previous reports [42, 43, 45–49] and Supplementary Figure S3. Biological age (BA) indicated by DNAmPhenoAge is colored according to the rank orders of cell type–specific group mean values for DNAmPhenoAge in GSE131989, GSE166844, GSE35069, and GSE78942 in this study (Table 2, Supplementary Table S2). Our results suggest a trend towards increasing numbers of cell types with “older” BA values with increasing chronological age; and vice versa, cell types with “younger” BA values decreasing with age.

So far, reports on blood cell type specificity in Horvath, DNAmPhenoAge, and DunedinPACE values have been based on pairwise comparisons between cell subtypes originating from different individuals [27] or small datasets (number of individuals < 10) [27], on whole blood data where cell proportions have been estimated using deconvolution methods [27, 50], or on a single BA indicator at a time [24, 26]. The strength of our approach was the inclusion of purified cell populations from four independent datasets, the application of s six BA indicators and the PC clocks, as well as the fact that the datasets comprised the same sets of individuals for each cell type. Furthermore, we were able to assess relationships between DNAmPhenoAge and DunedinPACE values and the donors’ chronological ages within a cell type for a larger number of individuals than in previous studies.

Our observations are in line with previous studies [24, 26, 27, 50], where comparable. In our analysis, subsets of T cells, especially naïve CD4 + and total CD8 + T cells, generally displayed the “youngest” values of the different BA indicators. Both CD19 + B cells and CD14 + monocytes displayed “older” BA indicator values than did T cells, and of the two, CD14 + monocytes displayed the “oldest” BA values. The differences between CD4 + CD28 + and CD4 + CD28- T cells were particularly pronounced. That is, naïve CD4 + T cells showed “younger” BA values than did memory CD4 + T cells, and CD4 + CD28 + T cells showed “younger” Horvath and DNAmPhenoAge values than did CD4 + CD28- T cells, and the differences amounted to up to 40 years.

We identified statistically significant differences for the Horvath pan-tissue clock [28] in the majority of pairwise comparisons in more than one independent dataset. These differences are in line with our previous findings [26] as well as other literature [24, 27, 50]. This finding is interesting, as this 1st generation clock was trained with data from a total of 53 somatic tissues [28] and one could expect different cell types to display similar values with this BA. In Kananen et al. (2016), Horvath values were higher with higher FACS analysis–based proportions of CD4 + CD28- T cells than of CD4 + CD28 + T cells, when assessed in cells originating from individuals of the same chronological age. The other previous studies have reported an up to twenty-year difference in Horvath values between different cell subtypes [24, 27, 50]. In our analysis, the group-level differences between the cell types were up to 9.5 years. Notably, the differences between whole blood sample and the other cell subtypes were generally smaller for Horvath than for Hannum, DNAmPhenoAge and DunedinPACE but larger than for DNAmTL and *ELOVL2* methylation.

Zhang et al. (2023) reported the lowest Hannum, Horvath, DNAmPhenoAge, and DunedinPACE values for naïve CD8 + T cells. Our datasets did not include naïve CD8 + T cells, only total CD8 + T cells, and they were generally observed to have lower BA values when compared to whole blood. In addition, in both datasets containing total CD8 + T cells, they showed the lowest BA values out of the different cell types. It is important to note that naïve T cells are more prevalent in blood than CD28- T cells, especially at younger chronological ages [44, 51], and we observed dramatic differences in their BA values when compared to whole blood while the BA values of CD4 + CD28- T cells are closer to those of whole blood. Thus, the magnitude of the possible contribution by naïve T cells to the BA values in a whole blood sample is substantial.

The DunedinPACE [31] values, when measured from whole blood, have been shown to increase with higher chronological age, even though this association is much weaker when compared to other epigenetic clocks [52]. Our correlation statistics from the dataset (GSE131989) with 49 blood donors indicate that the association between DunedinPACE and chronological age may not be the same in all cell types. In naïve CD4 + T cells, the Spearman’s correlation ρ was 0.64, whereas the correlation was weak or non-existent for memory CD4 + T cells, CD14 + monocytes, and CD19 + B cells (Spearman’s ρ < 0.3). This highlights the need for further studies on the effect of naïve CD4 + T cell counts on the DunedinPACE values measured from whole blood samples. For Of the other BA indicators (DNAmTL, methylation level at the *ELOVL2* CpG-site, as well as Hannum, Horvath, and DNAmPhenoAge), cell type–specific BA values correlated with chronological age strongly or very strongly (Spearman’s ρ > 0.7 or < -0.7).

The majority of our results show a similar direction and magnitude for pairwise comparisons in the different BA values between the cell types. Certain cell types were either “younger” or “older” according to most of the indicators—for example, naïve CD4 + T cells were very often “younger” than the other cells. The similarities might be explained by the fact that these BA indicators are based on DNA methylation, which is closely linked with cellular identity [53]. In parallel, according to some BA indicators, such as DNAmPhenoAge, monocytes are “older” than B cells and naïve and memory CD4 + T cells—but according to Hannum, they are not. The differences between the BA indicators may be explained by the fact that the different BA indicators represent different domains of biological aging (e.g., DNA methylation in a gene vs telomere length vs epigenetic clocks) and, of course, utilize varying sets of DNA methylation sites in the genome. Furthermore, the epigenetic clocks can also be categorized into generations depending on the building strategy. The 1st generation epigenetic clocks, such as the Horvath [28] and Hannum clocks [29], were built to predict chronological age; the 2nd generation epigenetic clocks, such as DNAmPhenoAge [30], were built to predict biological age utilizing biomarkers and chronological age; while the 3rd generation clock DunedinPACE [31] was built to predict the pace of aging, utilizing longitudinal biomarker and health data, and not chronological age as such. Horvath was trained with blood and multiple tissues [28], and the rest are only based on measurements from blood samples.

The significance of cell proportion for epigenetic ages has been noted, to some extent, in previous literature and is an important consideration for the concepts of intrinsic and extrinsic epigenetic ages [54]. These measures of biological aging are both residual values of an epigenetic clock, such as Horvath or Hannum, after adjusting for chronological age, but intrinsic epigenetic age aims to be independent of blood cell composition as the composition is adjusted for. However, for the extrinsic epigenetic age, the cell composition is incorporated into its values as an additive element. Thus, extrinsic age is not intended to be a measure of the deep cellular mechanism in the aging process, but it is a composite measure. In a meta-analysis of 13 cohorts by Chen et al. (2016), extrinsic age values resulted in a higher hazard ratio for mortality, with more narrow confidence intervals, than intrinsic age [54]. This implies that cell counts may yield additive value for lifespan prediction, for example, and that the cell composition is not solely a potential confounding factor.

DNA methylation–based BA indicators are often developed for and measured from whole blood or PBMC samples. They can be used in trials or interventions targeted at rejuvenation or the reversal of biological aging, but they can also be used to study physiological or pathological conditions not directly related to aging as such. As aging and various other physiological or pathological conditions can be simultaneously and independently associated with both changes in cell composition and increased epigenetic ageing, great care should be taken to disentangle the two. For example, a physically active lifestyle has been reported to rejuvenate the immune system by increasing the numbers of naïve T lymphocytes or by altering the CD4/CD8 ratio [55]. Conditions such as HIV and Parkinson’s disease have both been associated with increased epigenetic ageing and changes in cell composition [25, 56].

Fahy et al. (2019) have reported the reversal of epigenetic aging in PBMCs, indicated by four different epigenetic clocks, after a thymus-regenerating treatment [57]. The treatment resulted also in changes in cell composition. The authors report a decrease in monocytes and an increase in lymphocyte-to-monocyte ratio, decrease in PD-1 positive CD8 + cells, increase in naïve CD4 + and naïve CD8 + cells and an increase in CD4 + recent thymic emigrants. In an analysis adjusted for lymphocyte count, lymphocyte-to-monocyte ratio and percentage of senescent CD8 + cells, the decrease in values of epigenetic clocks by treatment remained statistically significant. However, the analyses were not adjusted for any CD4 T cell subsets. As our results show “younger” BA values for naïve CD4 + T cells, shown to be increased due to the thymus-regeneration treatment, it would be interesting to know the contribution of this cell type to the overall decrease in epigenetic ageing in the study. In other studies on potential aging interventions, cell proportions have not been taken into account [58], or only the baseline cell proportions have been accounted for [59]. In general, when interpreting the results of potential aging or health interventions, great care should be taken to define what is meant by rejuvenation and what it aims for. Is the objective to change the cells’ intrinsic processes or not? One might ask whether a change in immune cell proportions alone is a sufficient outcome for an intervention to be considered successful.

As an example of a physiological condition, it has been recently reported that pregnancy is associated with increased biological age, and that this increase is reversible postpartum [60, 61]. Pregnancy is associated with reversible changes in blood cell composition, with changes in both the total number and proportions of different cell types [62–64]. In the analysis by Pham et al. (2024), adjusting the statistical models with estimated cell proportions attenuated the association between biological age and the course of pregnancy. However, not all potentially relevant blood cell subtypes were accounted for in the analysis, and it would be interesting to see replication of this analysis with measured, instead of estimated, blood cell proportions (see Limitations and future perspectives).

In an analysis of genome-wide DNA methylation levels in mixed cell samples, upon which the epigenetic clocks are also based on, adjusting for cell type composition has been recommended [65], but also cautioned against [66]. Our results highlight the need for further analyses on this topic as they show that none of the DNA methylation-based BA indicator are fully immune to differences in cell proportions.

Interestingly, the results of our study also suggest that cell type-specificity is more evident for clocks that are ‘better’ predictors of health and/or lifespan. In our results, DunedinPACE and DNAmPhenoAge showed the most numerous or largest statistically significant pairwise differences across cell types, whereas differences across cell types were more modest for *e.g.* Horvath and DNAmTL. In previous studies DunedinPACE and DNAmPhenoAge have been shown to outperform Horvath and DNAmTL as predictors of morbidity and mortality [8, 30, 31]. However, a BA indicator with considerable cell type variability is not necessarily a particularly good BA indicator, as Hannum showed variability comparable to DunedinPACE, yet DunedinPACE has been shown to outperform Hannum [31]. More research is needed to disentangle whether cell-type variability is an inherent property of a good DNA methylation-based BA indicator and whether these two phenomenon can be untangled.

### Limitations and future perspectives

We show extreme and abundant differences in the values of ten BA indicators between the blood cell subtypes using four independent datasets. Importantly, we are able to demonstrate that the differences between the cell types appear to persist during adulthood, except for DunedinPACE. While our analysis included 1st, 2nd and 3rd generation epigenetic clocks as well as other DNA methylation based BA indicators, we did not include all the clocks that have been described in the literature [67–70]. These results, together with the knowledge on the wide ranges of and age-associated changes in cell subtype proportions at population level (Fig. 4), highlight the need for additional efforts when using the existing epigenetic clocks or building new ones. The cell composition in the blood samples may be accounted for in the statistical analysis, if the composition is measured, but measured cell type proportions are rarely available in large human cohort studies. One solution is to estimate the cell counts in a tissue sample using DNA methylation reference libraries for the various cell subtypes [71–73]. However, this cell count estimation is limited in two ways. Firstly, DNA methylation–based cell count estimates may show only modest correlations with the cell counts obtained using other DNA methylation–based estimation algorithms [74], and the reliability of the cell count estimation algorithms should be further evaluated in relation to, for example, FACS-based cell counts in larger, independent population cohorts. Secondly, current libraries do not cover all of the different blood cell subtypes with diverse functionalities, such as the more specific CD4 + T cell subpopulations [75] including regulatory T cells [76], or various B cell [77] or NK cell [78] subpopulations. For example, NK cell subtypes show drastic changes in their abundance and/or functionality/properties with aging and/or age-related pathologies [79]. This limitation also extends to our analysis. Even though our observations are from sets of purified cell types that are often considered to represent “detailed cell separation” (Fig. 4), many potentially relevant blood cell subtypes could not be analyzed in our study because DNA methylation data are not available for the cell types. Overall, our results highlight the need for analyses on the BA indicators in single cells.

In addition, even when the cell separation protocols and purity levels are in accordance with the high standards in the field, cell subsets are hardly ever completely purified. In the four datasets used in this study, cells were separated using varying FACS protocols, and, for example, a cell subtype was sometimes determined with only one surface antigen and sometimes by using more than one (Supplementary Table S1). The impurity may have influenced our results and caused noise in the cell subtype–specific BA values. The consistency of our findings suggests that the extent of this noise is likely small, but further studies are needed.

## Conclusions

Different blood cell subtypes generally show distinct biological ages (BAs), according to six BA indicators representing various aspects of biological aging. The magnitude of difference between whole blood cells and a blood cell subtype can be substantial, up to 160%, and the magnitudes are on average 20 percent or more for Hannum, DNAmPhenoAge and DunedinPACE, 10 percent for Horvath and less than 10 percent for DNAmTL and *ELOVL2* methylation. The differences appear to persist across adult ages from 20 to 80 years for all BA indicators, with the exception of DunedinPACE. When studying DNA methylation–based BA indicators in whole blood samples, the contribution of differing blood cell proportions needs to be considered. Additionally, the relative contributions of cell composition changes, epigenetic maintenance mechanisms and other potential mechanisms to variation in BA indicators’ values warrants further research. These information are relevant for studies on physiological and pathological conditions known to have a significant effect on blood cell proportions, but especially for any potential aging interventions.

## Supplementary Information

Below is the link to the electronic supplementary material.

Supplementary file1 (XLSX 66 KB)

Supplementary file2 (DOCX 1779 KB)
