# Supplementary material for: Biological aging of different blood cell types
Source: GeroScience. 2024 Jul 26;47(1):1075–92. doi: 10.1007/s11357-024-01287-w (PMC11872950; doi:10.1007/s11357-024-01287-w)
Supplement: Supplementary file 2 — Supplementary file2 (DOCX 1779 KB) [file 11357_2024_1287_MOESM2_ESM.docx]

**Biological aging of different blood cell types**

Saara Marttila^1,2,3*^, Sonja Rajić^1^, Joanna Ciantar^1^, Jonathan KL Mak^4,5^, Ilkka S Junttila^6-9^, Laura Kummola^6^, Sara Hägg^4^, Emma Raitoharju^1,3^, Laura Kananen^2,4,10,11^*

**Affiliations**

1 Molecular Epidemiology (MOLE), Faculty of Medicine and Health Technology, Tampere University, Tampere, Finland

2 Gerontology Research Center, Tampere University, Tampere, Finland

3 Tays Research Services, Wellbeing Services County of Pirkanmaa, Tampere University Hospital, Tampere, Finland

4 Department of Medical Epidemiology and Biostatistics, Karolinska Institute, Stockholm, Sweden

5 Department of Pharmacology and Pharmacy, Li Ka Shing Faculty of Medicine, The University of Hong Kong, Hong Kong, China

6 Faculty of Medicine and Health Technology, Tampere University, Tampere, Finland

7 Fimlab Laboratories, Tampere, Finland

8 Northern Finland Laboratory Centre (NordLab), Oulu, Finland

9 Research Unit of Biomedicine, University of Oulu, Oulu, Finland

10 Faculty of Social Sciences (Health Sciences), Tampere University, Tampere, Finland

11 Department of Neurobiology, Care Sciences and Society (NVS), Karolinska Institute, Stockholm, Sweden

* Corresponding authors:

Marttila Saara, [saara.marttila@tuni.fi](mailto:saara.marttila@tuni.fi)

Kananen Laura, [laura.kananen@ki.se](mailto:laura.kananen@ki.se), [laura.kananen@tuni.fi](mailto:laura.kananen@tuni.fi)

- **Supplementary Figures S1–S3**
- **Supplementary Results**


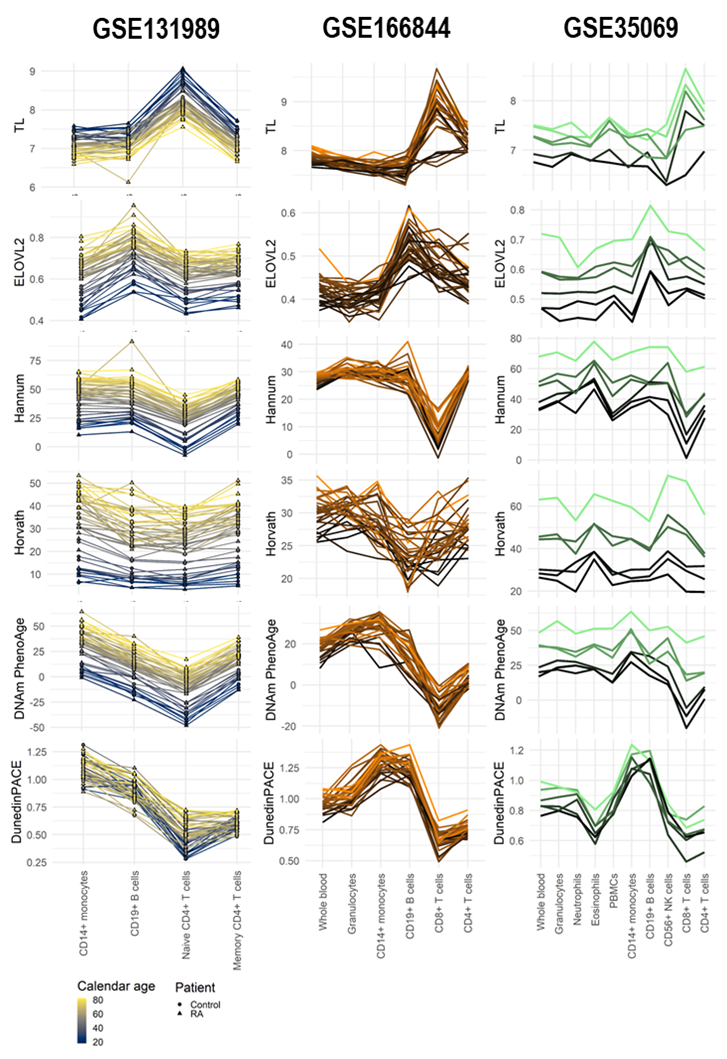


**Figure S1.** **Blood cell type–specific biological ages (BAs) and a BA rate**

DNA methylation–based indicators were assessed in three DNA methylation datasets (GSE131989, GSE166844, GSE35069), with 424 biological samples from 83 individuals and including 12 cell sample types. Lines are colored according to chronological age for GSE131989, and according to BA indicator values in whole blood samples for GSE166844 and GSE35069 because chronological age was either constant or not available. Each line represents one individual.

**
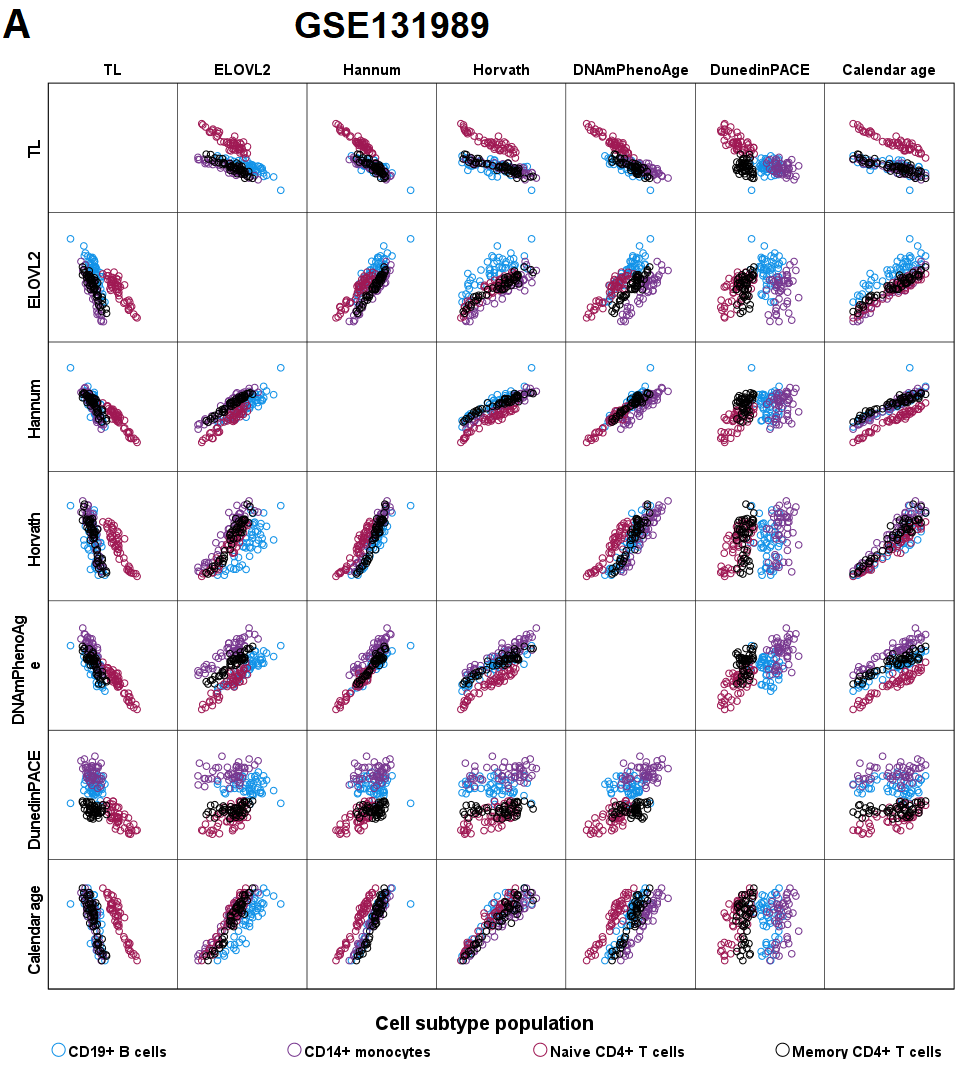

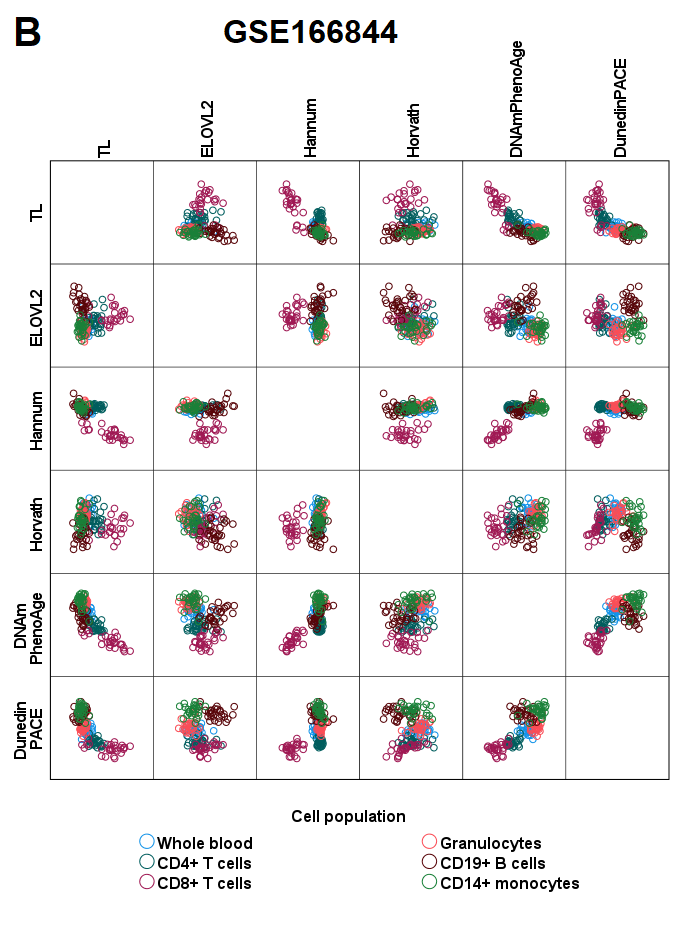
**

**
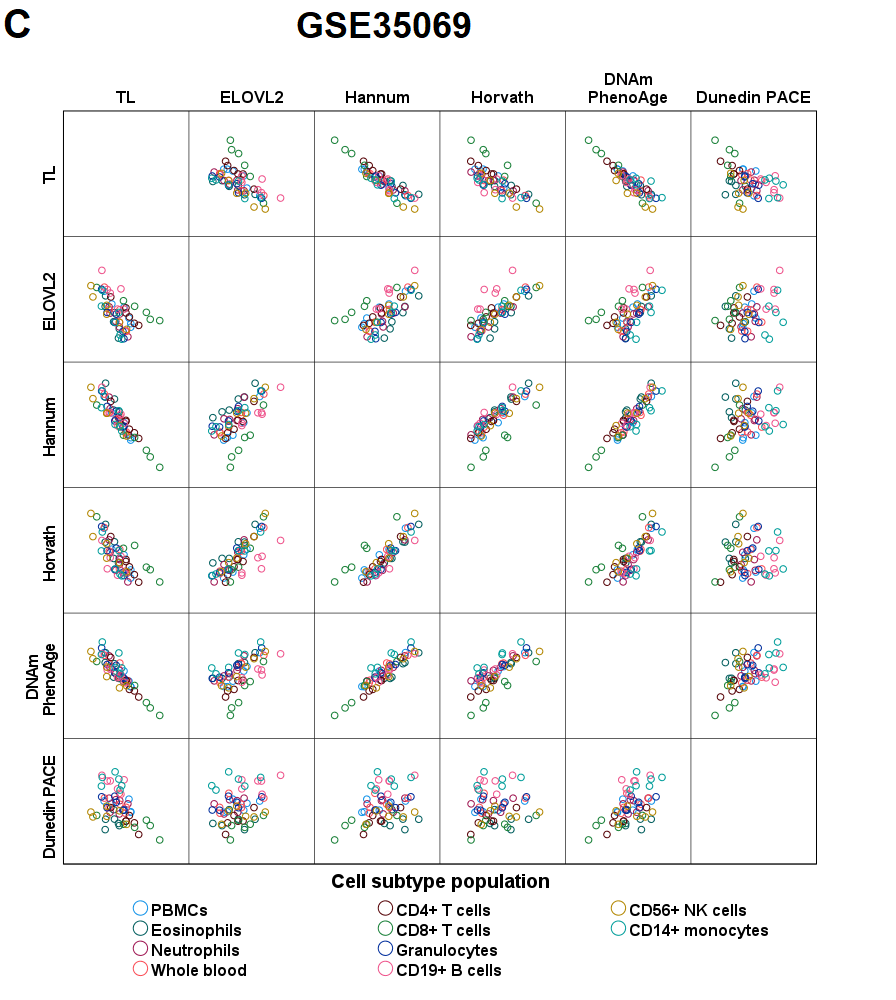
**

**Supplementary Figure S2. Relationship between the absolute values of the six BA indicators and chronological age in the three data sets, GSE131989 (A), GSE166844 (B), and GSE35069 (C)**

In each panel (A-C), pairwise relationships in each dataset are visualized as scatterplots. Thus, in panels A, B, and C, there are 196, 168, and 60 biological samples, respectively, which are colored according to the cell subtype population. Correlation coefficients within each cell subtype population are shown in Supplementary Table S8-10.


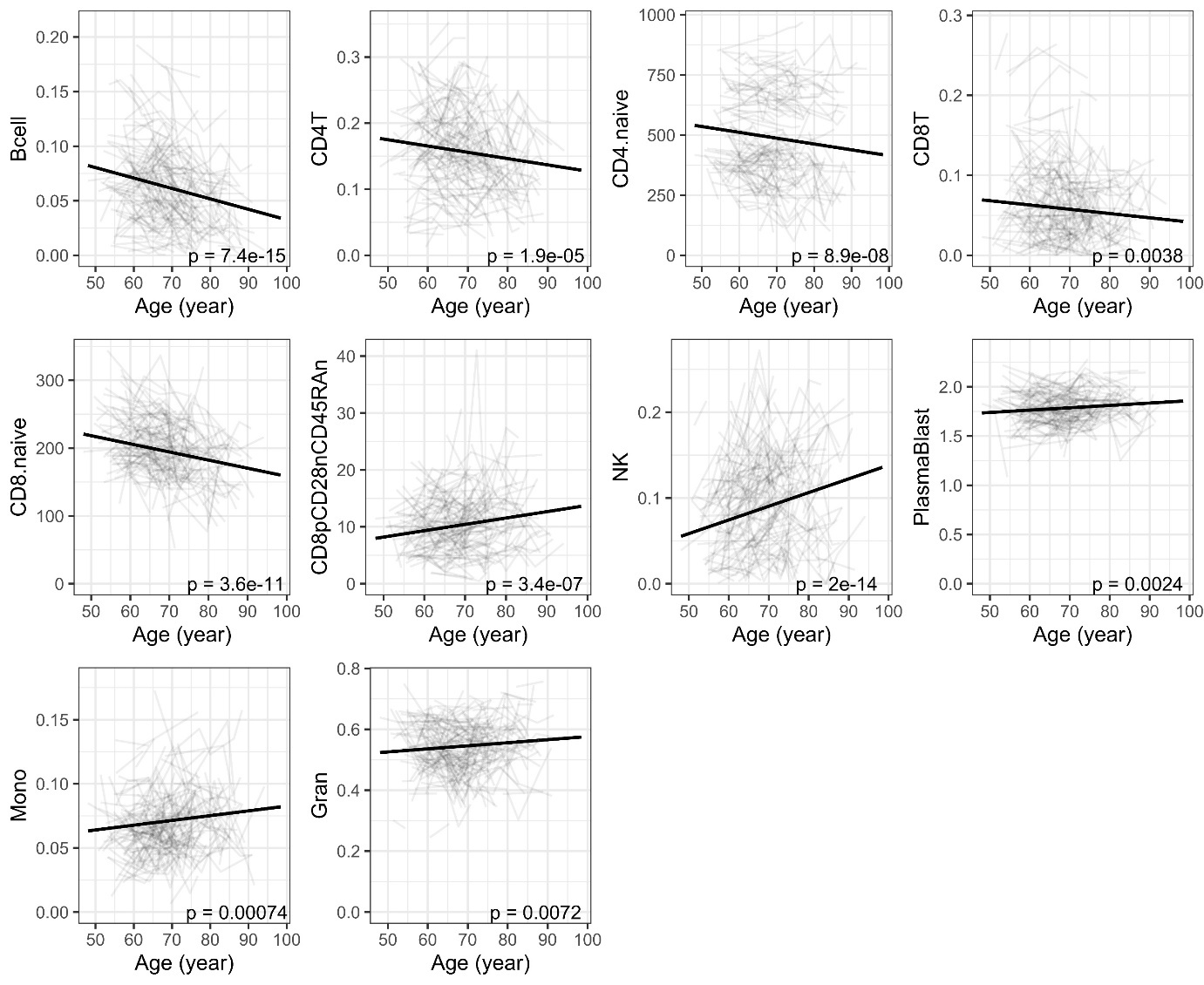


**Supplementary Figure S3. Cell count trajectories in blood with advancing calendar age**

Data are from a longitudinal cohort (SATSA, n= 328). p-value is for yearly change from a linear mixed model analysis. Cell counts were estimated from DNA methylation data (Wang et al., 2018). For this analysis, outlier values were rigorously filtered out from the cell count data (excluding values 0).

**Supplementary results**

*Detailed description of the pairwise comparisons between cell types and whole blood*

*CD19+ B cells*

Methylation data for CD19+ B cells were available in three datasets. When compared to other cell types, CD19+ B cells displayed a statistically significant difference (Mann–Whitney U test p-value < 0.05) in the majority of the pairwise comparisons in GSE131989 and GSE166844 (Figure 2, Supplementary S3–S4). In the smallest dataset, GSE35069, statistically significant differences were mainly observed with DunedinPACE (Figure 2, Supplementary Figure S1, Supplementary Table S5). In summary, our results suggest that CD19+ B cells are, according to the studied BA indicators, “younger” than CD14+ cells, but “older” than naïve CD4+ cells and total CD8+ T cells, although there are some discrepancies between the different BAs (Figure 2, Supplementary Table S3–S5). In comparison to whole blood, no clear pattern was observed for CD19+ B cells.

When comparing CD19+ B cells with CD14+ monocytes, lower Horvath, DNAmPhenoAge, and DunedinPACE values were detected in GSE131989 and GSE166844, suggesting that CD19+ cells are “younger” than CD14+ monocytes. In contrast, *ELOVL2* methylation was higher for CD19+ B cells than for CD14+ monocytes in the GSE35069 and GSE131989 datasets, suggesting that they are “older”, as *ELOVL2* hypermethylation has been shown to be associated with aging (Garagnani et al., 2012) (Figure 2, Supplementary Tables S3–S5).

When comparing CD19+ B cells with CD4+ naïve T cells, we observed a concordant pattern across different BA indicators, suggesting that CD19+ B cells are “older” than CD4+ naïve T cells. CD19+ B cells had higher DNAmHannum, DNAmPhenoAge, DunedinPACE, and *ELOVL2* methylation values, and a lower DNAmTL value than did CD4+ naïve T cells. When comparing CD19+ B cells to CD4+ memory T cells, a similar pattern of “older” values for CD19+ B cells can be observed for DunedinPACE and *ELOVL2* methylation, both of which are higher for CD19+ B cells than for CD4+ memory cells. In contrast, CD19+ B cells showed a “younger”, i.e. lower, Horvath value when compared to CD4+ memory T cells (Figure 2, Supplementary Table S3).

When comparing CD19+ B cells with total CD4+ T cells, four out of the six BA indicators suggested CD19+ cells to be “older” than total CD4+ T cells, whereas two of the six BA indicators suggested CD19+ B cells to be “younger” than total CD4+ T cells. “Older” BAs for CD19+ B cells in comparison to total CD4+ T cells were suggested by higher DunedinPACE values and *ELOVL2* methylation and lower of DNAmTL values in two datasets (GSE35069 and GSE166844) and by higher DNAmPhenoAge values in one dataset (GSE166844). In contrast, “younger” BA values for CD19+ B cells as opposed to total CD4+ T cells were suggested by lower Horvath and Hannum values (Figure 2, Supplementary Tables S4 and S5).

When comparing CD19+ B cells with total CD8+ T cells, we observed a clear pattern across different BA indicators, suggesting that CD19+ B cells are “older” in comparison to total CD8+ T cells. CD19+ B cells had higher Hannum, DNAmPhenoAge, DunedinPACE, and *ELOVL2* methylation values and a lower DNAmTL value than did total CD8+ T cells in GSE166844, and this was replicated with the Hannum and DunedinPACE values in GSE35069. The only contrasting BA indicator was Horvath, for which CD19+ B cells showed a lower value in GSE166844 when compared to total CD8+ T cells (Figure 2, Supplementary Tables S4, S5).

When comparing CD19+ B cells with whole blood samples, there was no clear pattern regarding the direction of difference. Two of the BA indicators suggested CD19+ B cells to be “younger” than whole blood, as the Horvath and DNAmPhenoAge values were lower for CD19+ B cells than for whole blood in GSE166844. However, three of the BA indicators suggested CD19+ B cells to be “older” than whole blood, as the DunedinPACE values and *ELOVL2* methylation levels of CD19+ B cells were higher in GSE166844 and GSE35069 and the DNAmTL value was lower in GSE166844 in comparison to those of whole blood (Figure 2, Supplementary Tables S4, S5).

*T cell subsets*

Data on various subsets of T cells were available in four datasets, including total CD4+ and CD8+ T cells (GSE166844 and GSE35069), CD4+ naïve and memory T cells (GSE131989), and CD4+CD28- and CD4+CD28+ T cells (GSE78942). The majority of pairwise comparisons across these cell types were statistically significant (Figure 2, Table 2, Supplementary Tables S3–S5). Our results suggest that CD8+ T cells are “younger” when compared to CD4+ T cells and that naïve CD4+ T cells are “younger” than memory CD4+ T cells (Figure 2, Supplementary Table S3–S5). In addition, CD4+CD28+ cells were identified to be “younger” than CD4+CD28- according to both BA indicators available for this dataset, Horvath and DNAmPhenoAge (Table 2), although no statistical tests could be performed on these data, as there were only four biological samples. When compared to whole blood, both CD4+ and CD8+ T cells are “younger”, although there are discrepancies between different BA indicators (Figure 2, Supplementary Table S3–S5). The magnitude of the difference was larger between CD8+ T cells and whole blood than between CD4+ T cells and whole blood (Figure 1, Figure 2).

When comparing CD8+ T cells to CD4+ T cells, a clear pattern could be observed, suggesting that CD8+ T cells are “younger” than CD4+ T cells. The Horvath, Hannum, DNAmPhenoAge, and DunedinPACE values were lower and the DNAmTL values were higher for CD8+ T cells than for CD4+ T cells (GSE166844, Figure 2, Supplementary Table S4). In GSE35069 the difference between CD8+ and CD4+ T cells was not statistically significant, but a similar trend could be observed for Hannum and DNAmPhenoAge (Figure 2). In contrast, *ELOVL2* methylation levels suggested CD8+ T cells to be “older” than CD4+ T cells, as the value of this BA indicator was higher for CD8+ T cells (GSE166844, Figure 2, Supplementary Table S4).

When comparing CD4+ naïve T cells to CD4+ memory T cells, we observed a concordant pattern, suggesting that CD4+ naïve T cells are “younger” than CD4+ memory T cells. The Horvath, Hannum, DNAmPhenoAge, and DunedinPACE values were lower and DNAmTL values higher for CD4+ naïve T cells than for CD4+ memory T cells (GSE131989, Figure 2, Supplementary Table S3). Another CD4+ T cell subset for which we had data available were CD4+CD28+ and CD4+CD28- cells (GSE78942). As this dataset consisted of only 4 biological samples, no statistical test could be performed. However, CD4+CD28+ cells were identified to be “younger” in comparison to CD4+CD28- according to both BA indicators, Horvath and DNAmPhenoAge, available in the dataset (Table 2).

When comparing the different T cell populations to whole blood, we observed a clear pattern suggesting that CD8+ T cells are “younger” compared to whole blood samples. The Horvath, Hannum, DNAmPhenoAge, and DunedinPACE values were lower and the DNAmTL value was higher for CD8+ T cells when compared to whole blood (GSE166844, Figure 2, Supplementary Table S4). A similar pattern could be observed for Hannum and DunedinPACE in the small dataset GSE35069 (Figure 2, Supplementary Table S5). However, *ELOVL2* methylation levels in GSE166844 suggested an opposite pattern of CD8+ T cells being “older” than whole blood, as the value of this indicator was higher for CD8+ T cells (Figure 2, Supplementary Table S4).

When comparing CD4+ T cells to whole blood, the results were similar to those for CD8+ T cells. Based on four of the six BA indicators, CD4+ T cells were suggested to be “younger” than whole blood. The Horvath, DNAmPhenoAge, and DunedinPACE values were lower and the DNAmTL value was higher for CD4+ T cells than for whole blood (GSE166844, Figure 2, Supplementary Table S4). A similar pattern was observed for DunedinPACE in GSE35069 (Figure 2, Supplementary Table S5). As regards CD4+ T cells, two BA indicators suggested them to be “older” than whole blood, as the Hannum values and *ELOVL2* methylation levels were higher for CD8+ T cells than for whole blood (Figure 2, Supplementary Table S4). For the majority of these differences, the magnitude of the difference was larger between CD8+ T cells and whole blood than between CD4+ T cells and whole blood (Figure 1, Figure 2).

*CD14+ monocytes*

Data on CD14+ monocytes were available in three datasets. When compared to other cell types, the majority of the pairwise comparisons between CD14+ monocytes were statistically significant in GSE166844 and GSE131989 (Figure 2, Supplementary Figure S1, Supplementary Tables S3 and S4). The prominent differences between CD14+ monocytes and CD19+ B cells have been described in detail in the previous section. Our results suggest that CD14+ monocytes are “older” in comparison to various T cell subsets as well as whole blood samples (Figure 2, Supplementary Tables S3–S5).

When comparing CD14+ monocytes to CD8+ T cells, we observed a clear pattern suggesting that CD14+ monocytes are “older” than CD8+ T cells. The Horvath, Hannum, DNAmPhenoAge, and DunedinPACE values were higher and the DNAmTL value was lower for CD14+ monocytes than for CD8+ T cells in GSE166844, and the same pattern was observed for Hannum, DNAmPhenoAge, and DunedinPACE values in GSE35069 (Figure 2, Supplementary Tables S4, S5). In contrast, the lower *ELOVL2* methylation level suggested CD14+ monocytes to be “younger” in comparison to CD8+ T cells (GSE166844, Figure 2, Supplementary Table S4).

When comparing CD14+ monocytes to CD4+ T cells, three of the studied BA indicators suggested CD14+ monocytes to be older. The values obtained with DNAmPhenoAge and DunedinPACE were higher and those obtained with DNAmTL were lower for CD14+ monocytes than for CD4+ T cells in two datasets (GSE166844 and GSE35069, Figure 2, Supplementary Tables S4, S5). Only the lower rate of *ELOVL2* methylation suggested CD14+ monocytes to be “younger” than CD4+ T cells (GSE166844, Figure 2, Supplementary Table S4).

When comparing CD14+ monocytes to naïve and memory subsets of CD4+ T cells, a pattern similar to the one detected with CD4+ T cells can be observed, which is to say that CD14+ cells are “older”. The Horvath, Hannum, DNAmPhenoAge, and DunedinPACE values are higher and the DNAmTL value is lower for CD14+ monocytes than for CD4+ naïve T cells. DNAmPhenoAge and DunedinPACE values are higher for CD14+ monocytes than for CD4+ memory T cells (GSE131989, Figure 2, Supplementary Table S3).

When comparing CD14+ monocytes to whole blood, we observed a concordant pattern suggesting that CD14+ monocytes are “older” than whole blood samples. Hannum, DNAmPhenoAge, and DunedinPACE values were higher and the DNAmTL value was lower for CD14+ monocytes than for whole blood (GSE166844, Figure 2, Supplementary Table S4). For DunedinPACE, this finding was replicated in the GSE35069 dataset (Figure 2, Supplementary Table S5).

**References**

Garagnani, P., Bacalini, M. G., Pirazzini, C., Gori, D., Giuliani, C., Mari, D., Di Blasio, A. M., Gentilini, D., Vitale, G., Collino, S., Rezzi, S., Castellani, G., Capri, M., Salvioli, S., & Franceschi, C. (2012). Methylation of ELOVL2 gene as a new epigenetic marker of age. *Aging Cell*, *11*(6), 1132–1134. https://doi.org/10.1111/acel.12005

Wang, Y., Karlsson, R., Lampa, E., Zhang, Q., Hedman, Å. K., Almgren, M., Almqvist, C., McRae, A. F., Marioni, R. E., Ingelsson, E., Visscher, P. M., Deary, I. J., Lind, L., Morris, T., Beck, S., Pedersen, N. L., & Hägg, S. (2018). Epigenetic influences on aging: A longitudinal genome-wide methylation study in old Swedish twins. *Epigenetics*, *13*(9), 975–987. https://doi.org/10.1080/15592294.2018.1526028
